# Radiative Cooling: Principles, Progress, and Potentials

**DOI:** 10.1002/advs.201500360

**Published:** 2016-02-04

**Authors:** Md. Muntasir Hossain, Min Gu

**Affiliations:** ^1^ Centre for Micro‐PhotonicsFaculty of ScienceEngineering and TechnologySwinburne University of TechnologyHawthornVictoria3122Australia

**Keywords:** atmospheric radiation, radiative cooling, selective radiators, thermal radiation

## Abstract

The recent progress on radiative cooling reveals its potential for applications in highly efficient passive cooling. This approach utilizes the maximized emission of infrared thermal radiation through the atmospheric window for releasing heat and minimized absorption of incoming atmospheric radiation. These simultaneous processes can lead to a device temperature substantially below the ambient temperature. Although the application of radiative cooling for nighttime cooling was demonstrated a few decades ago, significant cooling under direct sunlight has been achieved only recently, indicating its potential as a practical passive cooler during the day. In this article, the basic principles of radiative cooling and its performance characteristics for nonradiative contributions, solar radiation, and atmospheric conditions are discussed. The recent advancements over the traditional approaches and their material and structural characteristics are outlined. The key characteristics of the thermal radiators and solar reflectors of the current state‐of‐the‐art radiative coolers are evaluated and their benchmarks are remarked for the peak cooling ability. The scopes for further improvements on radiative cooling efficiency for optimized device characteristics are also theoretically estimated.

## 1 Introduction

Passive cooling systems offer substantial impact as energy saving devices due to their ability to operate without external energy. Radiative cooling is such a promising cooling method with their ability to offer a cooling power of more than 100 W m^−2^ with optimized device designs and under suitable atmospheric conditions.^1^, ^2^ The potential of this passive cooling method for energy saving applications has also been realized.^3^, ^4^, ^5^, ^6^ The key behind this unmatched ability stems from releasing energy via the radiative heat exchange where the heat is dumped directly to the outer space. The significance of radiative cooling and its potential application was emphasized a few decades ago and practical cooling during the nighttime operation was also demonstrated.^7^, ^8^, ^9^, ^10^, ^11^, ^12^, ^13^, ^14^, ^15^, ^16^, ^17^, ^18^, ^19^, ^20^, ^21^, ^22^, ^23^ The use of bulk materials comprising intrinsic infrared (IR) emissions for considerable radiative cooling were discussed earlier.^10^, ^18^, ^21^, ^23^, ^24^ However, radiative cooling was mostly limited during the nighttime as suitable materials with high IR emission within the atmospheric window and yet delivering strong solar reflection during the day was not achieved. Although some solar reflecting materials were reported, daytime cooling below the ambient temperature was not achieved as the absorbed solar energy exceeded the emitted energy by thermal radiation.^25^, ^26^ It was only the recent demonstrations where the use of advanced nanophotonics led to daytime radiative cooling well below the ambient temperature.^3^, ^27^ The radiative coolers with photonic designs can simultaneously possess a high solar reflection up to 97% and strong IR emission within the atmospheric window.^1^, ^3^, ^27^ On the other hand, microstructure based thermal emitters can also offer highly efficient cooling power with their strictly selective and strong IR emission within the atmospheric window.^2^ These emerging photonic devices, offering substantial passive cooling during the day and night, have triggered significant research interest.

In this article, we seek to review the detailed characteristics, recent advancements, and the scope for further performance improvements of radiative coolers. In Section 2, we present a basic overview of operating principles of radiative cooling. The material and structural design requirements for nocturnal and daytime applications are identified and the potential cooling efficiency is investigated. The performances for selective and broadband radiators are compared. The impact of atmospheric conditions and nonradiative heat exchange processes on cooling efficiency is briefly discussed. In Section 3, we review the materials and device designs used for nocturnal radiative cooling in earlier studies. We analyze their performances and discuss their limitations for use in daytime applications. Section 4 discusses the recent developments of daytime radiative coolers based on photonic approach and microstructure based thermal emitters for daytime and highly efficient cooling applications. We also highlight the technical challenges for these devices for further performance improvement. In Section 5, the achievable cooling efficiency for optimized photonic and device characteristics are analyzed. Section 6 presents the conclusion of this article highlighting the passive cooling potentials of the radiative coolers.

## 2 Passive Cooling via the Atmospheric Window

**Figure 1:**
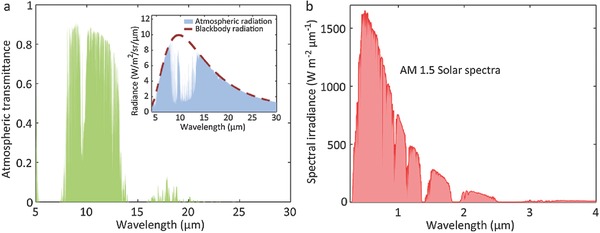
a) A modeled atmospheric transmittance (data from Ref. 28) with the downward atmospheric radiation in the inset. b) A reference AM 1.5 solar irradiance spectra (data from Ref. 30).

The Earth's atmosphere has a highly transparent window in the infrared (IR) wavelength range between 8 and13 μm, i.e., the atmosphere's radiative emission is very weak in that window. Outside the atmospheric window, the Earth's atmosphere is highly emissive. Coincidentally, the atmospheric window falls within the peak thermal radiation of a black body defined by Plank's law at the ambient temperature (at around 300 K). This feature permits for a passive cooling mechanism for a terrestrial body at the ambient temperature by eliminating heat via radiative emission through the atmospheric window. The emitted radiation escapes through the Earth's atmosphere to the outer space which acts as a gigantic thermal reservoir. The atmospheric window allows the terrestrial body's outgoing radiative emission to exceed its absorbed incoming atmospheric radiation and thus to passively cool below the ambient temperature. **Figure** **1**a shows the modeled atmospheric transmission^28^ in the zenith direction and the downward atmospheric radiation in a cloud‐free day for a typical mid‐latitude summer atmospheric conditions (Melbourne, Australia).^29^ Figure 1b shows the reference global AM1.5 solar irradiance spectra.^30^ The atmospheric window from 8 to 13 μm is clearly visible. A secondary atmospheric window also exists between 16 and 23 μm; however, it is fairly weak and its effect on radiative cooling can be neglected in general.

Along with the effect of the incoming atmospheric radiation, the cooling performance of a radiator depends on other factors, such as, the nonradiative (conductive and convective) heat gain from the surrounding media and the incoming solar radiation during the daytime. In addition, the cooling efficiency can heavily be affected by the emission profile of the radiator in use, i.e., a broadband radiator or a selective radiator. The IR window transmittance for certain atmospheric conditions can also significantly influence the cooing potential. The following subsections discuss the theoretical aspects of a radiative cooler and the associated factors affecting the cooling efficiency.

### 2.1 Principles of Radiative Cooling

Taking into account all the heat exchange processes, the net cooling power of a radiative cooler can be defined as

(1) $$P_{\mathrm{net}}=P_{r}-P_{a}-P_{\mathrm{nonrad}}-P_{\mathrm{sun}}$$

where

(2) $$P_{r}=2\pi \int_{0}^{\pi /2}\sin\theta \;\cos\theta \;d\theta \int_{0}^{\infty }U_{B}\left(T_{r},\lambda \right)\;e_{r}\left(\lambda ,\theta \right)\;d\lambda$$

is the radiative power emitted by the radiator and

(3) $$P_{a}=2\pi \int_{0}^{\pi /2}\sin\theta \;\cos\theta \;\;d\theta \int_{0}^{\infty }U_{B}\left(T_{a},\lambda \right)\;\;e_{r}\left(\lambda ,\theta \right)\;\;e_{a}\left(\lambda ,\theta \right)d\lambda$$

is the amount of the incident atmospheric radiation which is absorbed by the radiator.

Here, $U_{B}(T,\lambda )=\frac{2hc^{2}}{\lambda^{5}}\frac{1}{e^{hc/\lambda k_{B}T}-1}$ is the spectral radiance of a black body defined by Planck's law at any temperature *T*, where *h* is Planck's constant, *k*_B_ is the Boltzmann constant, *c* is the speed of light in vacuum, and *λ* is the wavelength. The emissivity of the radiator can be defined by its absorptivity $e_{r}(\lambda ,\theta )$ according to Kirchhoff's law. The angle dependent emissivity of the atmosphere is given by^21^ $e_{a}(\lambda ,\theta )=1-t{\left(\lambda \right)}^{1/\cos\theta }$, where *t*(*λ*) is the atmospheric transmittance in the zenith direction. *T*_r_ is the temperature of the radiator and *T*_a_ is the ambient temperature.

The third term of the right hand side of Equation (1) is the nonradiative (i.e., conductive + convective) heat gain of the radiator with the surrounding media, which can be defined as^1^, ^3^, ^25^, ^31^

(4) $$P_{\mathrm{nonrad}}=q\left(T_{a}-T_{r}\right)$$

where, $q=q_{\mathrm{cond}}+q_{\mathrm{conv}}$ is the combined nonradiative heat coefficient stemming from the conductive and convective heat exchange of the radiator with the surrounding air. Without any insulation for the nonradiative heat gain, the cooling power of the radiator is limited. In most of the experimental demonstrations, the radiators were insulated by polystyrene foam to suppress the conductive heat gain from the back surface and side walls and highly IR transparent low‐density polyethylene film was used as convection cover on top of the radiator with sufficient air gap.^3^, ^10^, ^12^, ^18^, ^31^, ^32^, ^33^ Nonradiative heat coefficient values ranging from 2 to 6.9 W m^−2^ °C^−1^ were estimated in several reports for radiative cooling which indicate effective suppression of nonradiative heat gain.^3^, ^12^, ^33^

The last term on the right hand side of Equation (1) expresses the absorbed solar power by the radiator which must be counted for estimating the radiative cooling efficiency during the daytime. The global AM1.5 solar spectrum has an irradiance of 1000 W m^−2^ and a significant absorption of this amount of power can easily diminish any cooling effect of the radiator. To avoid the adverse effects of solar radiation, IR transparent solar reflectors can either be integrated on the top of the thermal radiators or suitable cooling devices can be designed to serve as a simultaneous solar reflector and an IR radiator. The recent demonstrations of the daytime radiative coolers utilized the latter, where the cooling device reflected 97% of the incident solar radiation while providing significant IR emission within the 8–13 μm wavelengths range.^3^, ^27^

**Figure 2:**
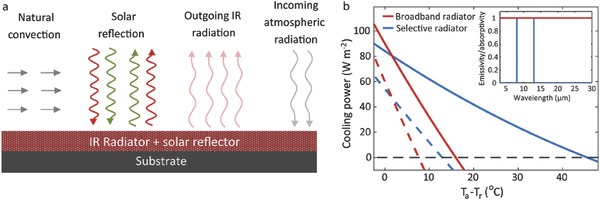
a) Schematics of a radiative cooler with some of the radiative and nonradiative processes. b) The cooling power and *T*_a_−*T*_r_ of a broadband and selective radiators. The solid curves represent the cooling with the exclusion of any solar and nonradiative contributions. The dashed curves include 3% solar power absorption and a practical nonradiative heat gain coefficient of 2 W m^−2^ °C^−1^. The intersections of the black dashed curve with the cooling power curves represent the values of *T*_a_−*T*_r_. Inset shows the spectral emissivity of an ideal selective and broadband radiators.

A radiator can provide with practical cooling only when the radiated output power exceeds the net absorbed power. With a positive value of *P*_net_ at the ambient temperature *T*_a_ in Equation (1), the radiator temperature *T*_r_ drops below *T*_a_ and reaches a steady state temperature *T*_ss_, (*T*_r_ = *T*_ss_ < *T*_a_) where the outgoing power balances the absorbed incoming power, i.e., *P*_net_ (*T*_ss_) = 0. Thus, to achieve a state where *T*_ss_ is significantly below *T*_a_, the radiative emission within the 8–13 μm wavelength region must be maximized and the absorbed power from the incoming atmospheric radiation, nonradiative contributions, and absorption of solar power must be minimized. **Figure** **2**a depicts a schematic diagram for a practical radiative cooling system.

### 2.2 Broadband and Selective Radiators

The performance of radiative coolers is highly related to the spectral emissivity profiles of the different kinds of radiators. The first one is a broadband radiator which has emissivity like a blackbody within the entire emission band of the atmosphere except the main solar spectral band from 300 nm to 2.5 μm. The second one is a selective radiator comprising unity emissions only within the wavelengths of 8–13 μm, selectively spanning over the atmospheric window. The ability of the selective radiator for cooling below the ambient temperature is vastly superior to that of a broadband radiator. As a comparison, the cooling power and the achievable cooling temperatures (*T*_a_–*T*_r_) of selective and broadband radiators with and without the presence of solar and nonradiative contributions are shown in Figure 2b where *T*_a_ = 25 °C. A practical cooling device with 50 W m^−2^ cooling power at the ambient temperature and covering only a surface area of 50 m^2^ on the rooftop of a residential building would allow removing heat equivalent to a net cooling load of 15 kWh for a peak daytime between 11 am and 5 pm. An estimate of energy savings (in kWh) by radiative coolers on rooftop operating over the course of a year has also been reported in ref. [3].

**Figure 3:**
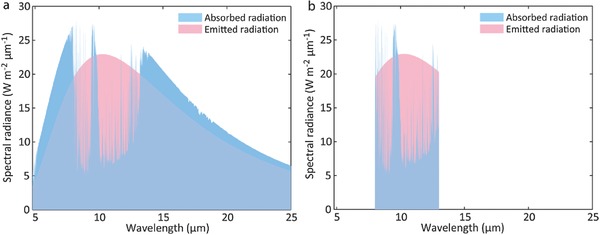
Absorbed atmospheric radiation and emitted radiation for a) broadband and b) selective radiators operating at 16 °C below *T*_a_.

The cooling abilities of radiators lie within their radiative emission profiles. A broadband radiator emits thermal radiation more than the incoming atmospheric radiation (outside the 8–13 μm window) and thus has a net cooling power. However, it may not be able to provide with a cooling temperature well below the ambient temperature (i.e., a high value of *T*_a_–*T*_r_) in practice. The reason behind is that a broadband radiator operating considerably below the ambient temperature, may emit the less radiative power than it receives from the incoming atmospheric radiation at the ambient temperature inhibiting it to cool further without any net cooling power. An ideal emitter thus should selectively and strongly emit within the entire 8–13 μm wavelengths range. This has important consequences. The emitter does not absorb radiation outside the 8–13 μm window where the atmosphere is highly emissive which allows a minimal radiative heat exchange with the atmosphere. The impact of this becomes highly important to achieve cooling significantly below the ambient temperature. In addition, a strong emission within the 8–13 μm window ensures a high emitted power to overcome the nonradiative and solar heat contributions to reach a steady state temperature significantly below the ambient temperature. To understand this further we consider the cooling powers of broadband and selective radiators in Figure 2b (without solar and nonradiative contributions) at a temperature 16 °C below the ambient temperature. At this temperature, the broadband radiator does not possess any net cooling power (Figure 2b, solid red curve). **Figure** **3** shows the amount of absorbed atmospheric radiation at *T*_a_ = 25 °C (integrated over the entire hemisphere) by the broadband and selective radiators (for the case in Figure 2b) and the emitted radiation at *T*_r_ = 9 °C. It can be clearly seen from Figure 3a that the emitted radiation by the broadband radiator at 16 °C below the ambient temperature balances the radiation it receives from the atmosphere at the ambient temperature and hence does not provide any effecting cooling (Figure 2b, the solid red curve). On the other hand, Figure 3b shows that the selective radiator receives minimal atmospheric radiation and still provides significant cooling power at 16 °C below the ambient temperature (Figure 2b, the solid blue curve). From Figure 2b, it is also evident that the absorption of the solar radiation and nonradiative heat gain processes greatly influence the cooling power of the radiators and decreases the gap of achievable cooling temperatures by the two types of radiators. This property is further discussed in Section 5.

Above the intersection point of the cooling powers for the broadband and selective radiators in Figure 2b, the blackbody radiator provides superior cooling power. This is due to the ability of a blackbody radiator to radiate over the broad spectral range where the total emitted power can far exceed the incoming atmospheric radiation, especially above the ambient temperature. For applications where a device itself produces a lot of heat or gains a lot of heat from other sources, the device temperature can reach high above the ambient temperature, where, radiative cooling below the ambient temperature may not be possible. In such a case, a broadband radiator can be more useful than a selective radiator to decrease the device temperature rather than cooling it below the ambient temperature.^34^, ^35^

### 2.3 Effects of Atmospheric Conditions

The effects of different atmospheric conditions on radiative cooling were discussed in several earlier reports.^36^, ^37^, ^38^ For atmospheric radiation, the atmospheric constituents like H_2_O, CO_2_, O_3_, CH_4_, and N_2_O contribute; however, for transmission within the atmospheric window, the major role is played by the H_2_O content within the atmosphere.^38^, ^39^ The amount of the water vapor content in the atmosphere can be associated to ground surface temperature and relative humidity (RH) or dew point.^37^, ^38^, ^39^ Here, we will briefly discuss the variation of the atmospheric transmittance for different locations and estimate the cooling potential for variable RH, assuming cloud‐free sky conditions.

**Figure 4:**
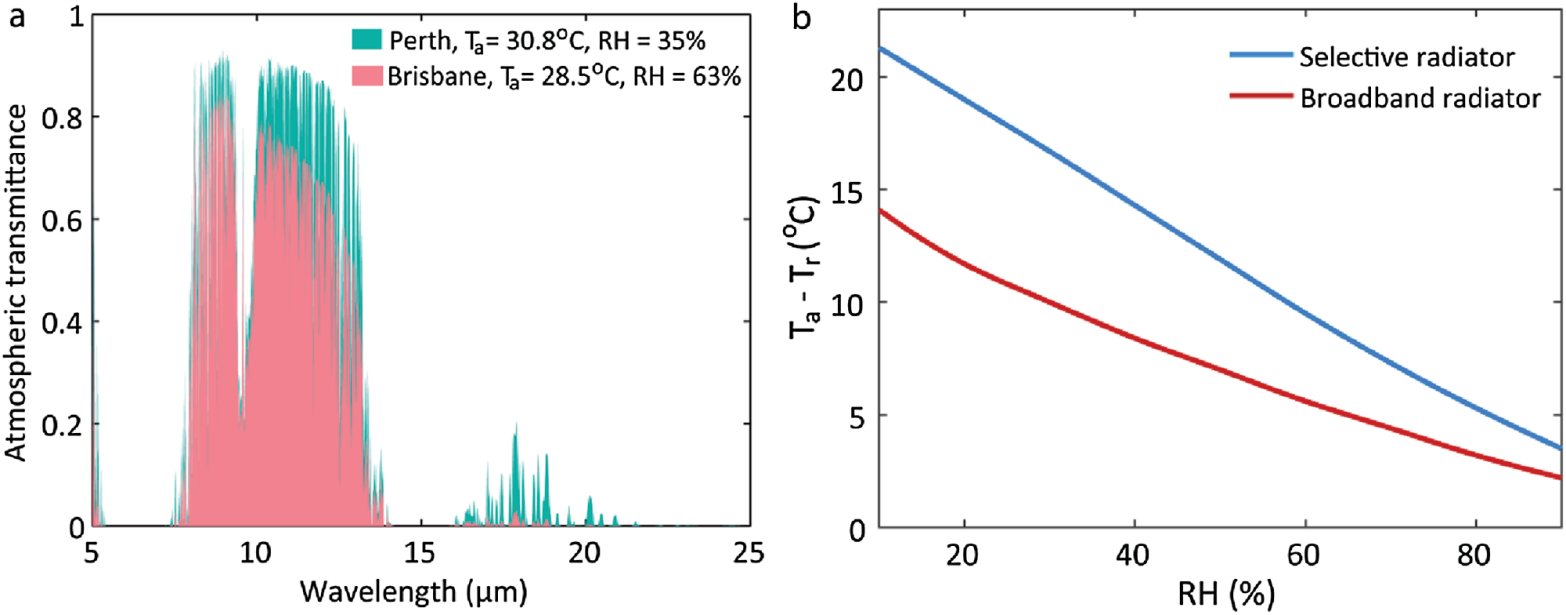
a) Modeled atmospheric transmittance profiles (data from Ref. 28) for atmospheric conditions in Perth and Brisbane. b) Calculated *T*_a_−*T*_r_ for a selective and broadband radiator with variable RH.

**Figure** **4**a shows the modeled atmospheric transmittance^28^ for the ambient temperatures and mean RH during the peak daytime (3 pm) at summer (January, 2015) in two mid‐latitude locations in Australia.^29^ It should be noted that although the peak temperatures are quite similar, the RH values of Perth and Brisbane vary by a significant margin and thus the atmospheric transmittance. To estimate the effect of humidity on the radiative cooling performance, we calculate the lowest achievable temperature below the ambient temperature, i.e., *T*_a_−*T*_r_ for a variable RH. For simplicity, the ambient temperature was set to 30 °C and the solar absorption and nonradiative contributions were set to practical values of 3% and 2 W m^−2^ °C^−1^. As can be seen from Figure 4b, effect of atmospheric water vapor on radiative cooling efficiency can be significant. For a selective and broadband radiator, *T*_a_−*T*_r_ drops from 21.3 to 3.5 °C and 14.1 to 2.2 °C. Another point to notice is that the difference of cooling abilities for selective and broadband radiators also decreases for increasing RH. In tropical climate zones, the humidity is high in general and meaningful cooling is only achievable at winter season, where a nighttime cooling of only 1–6 °C below ambient temperature was reported under clear sky conditions.^40^ Hence, the geographical location with certain atmospheric conditions and the choice of radiator types can play important role in practical radiative cooling applications. In addition, for a cloudy sky, the atmosphere will be completely opaque to IR radiation and any effective cooling will not be possible.

It has been known that the atmospheric radiation increases within the 8–13 μm wavelengths region for increasing zenith angle.^36^ Technical ways of blocking incoming atmospheric radiation to a radiator fully open to the sky were suggested by using reflectors or apertures with confining radiation to the sky into a small solid angle.^41^, ^42^ This leads to the prevention of incoming atmospheric radiation for large zenith angles, i.e., close to the horizontal directions.^36^, ^41^, ^42^

## 3 Conventional Approaches for Nighttime Radiative Cooling

Ever since the potentials of radiative cooling were realized, continuous efforts have been made to increase the cooling efficiency of the radiative emitters and practical cooling at night was also demonstrated.^7^, ^8^, ^9^, ^10^, ^11^, ^15^, ^16^, ^17^, ^18^, ^19^ However, the use of naturally available materials and synthetic polymers has always been the limiting factor. The possibilities of daytime radiative cooling were also discussed in different studies.^4^, ^25^, ^26^ The lack of suitable materials with high solar reflection but possessing strong emission within the IR window restricted any effective radiative cooling under the direct sunlight. For the realization of efficient nocturnal radiative cooling, selective IR emitters of various bulk materials have been studied. Among those, polymer films,^10^, ^12^, ^13^ white pigmented paints,^18^, ^32^ silicon monoxide (SiO) films, 20, 21, 22, 33 and other solid materials^43^, ^44^ have been widely investigated. Although these radiators provide somewhat selective radiation within the atmospheric transmission window, many of them suffer from weak emissivity, limiting the cooling performance. Furthermore, the lack of strict selectivity of the IR emission/absorption, results in significant absorption of the atmospheric radiation outside the transparency window, not allowing the cooling device to achieve steady state temperature significantly below the ambient temperature.^10^, ^12^, ^32^, ^33^

In this section, we will discuss the commonly used materials and devices for nighttime radiative cooling. We will highlight the IR emission properties and analyze their reported cooling performances.^2^, ^10^, ^18^, ^20^, ^21^, ^22^, ^23^, ^25^, ^26^, ^32^, ^45^, ^46^

### 3.1 IR Radiators

Catalanotti et al. and Bartoli et al. utilized a commercially available polyvinyl‐fluoride polymer film (TEDLAR) as the radiative emitter which possesses somewhat selective and high IR absorption from 9 to 13 μm wavelengths range.^10^, ^12^ However, significant IR absorption also occurs beyond 20 μm and multiple absorption peaks exist for wavelengths smaller than 8 μm.^13^, ^21^ This weak selectivity limits the radiative cooling efficiency of the polymer film. The authors demonstrated that by properly insulating their sample (10 × 10 cm^2^ of size) and using IR transparent polyethylene cover, the nonradiative heat transfer can be significantly minimized.^12^ A temperature reduction of 10 °C below the ambient was reported during the daytime but only under diffused sunlight conditions.^10^, ^12^ The advantage of the polymer not only lies in its intrinsic IR absorption but also in its ability to create large scale films for practical applications. However, the limitation of polyvinyl‐fluoride (PVF) polymer for radiative cooling during the day is due to its significant optical absorption within the solar spectra.^47^ Other types of polymer films have also been investigated for radiative cooling applications which include polyvinylchloride (PVC)^9^ and poly(4‐methylpentene) (TPX).^13^ However, the IR emissivity profiles of these polymers are worse than those of PVF in terms of selectivity and strong emission within the 8–13 μm range. PVC has high and broadband IR absorption for wavelengths outside the transparency window and TPX possesses IR absorption for wavelengths smaller than 8 μm, making them less suitable for efficient radiative cooling.

Apart from the polymer films, pigmented paints, naturally available inorganic compounds and gases with IR emission were also investigated for nocturnal radiative cooling applications.^18^, ^20^, ^21^, ^22^, ^24^, ^31^, ^32^, ^46^, ^48^, ^49^, ^50^, ^51^ The early demonstration of using pigmented paints reported significant cooling during the night under clear sky conditions.^18^ Commercially available white paint containing titanium dioxide (TiO_2_) was coated on aluminum plates and was used as the IR radiator. Although realistic cooling was not achieved directly under the sun at mid noon, a steady cooling of around 10 °C below the ambient temperature under clear sky conditions was reported during the night. On a relatively low humid day a maximum cooling 15 °C below the ambient was also demonstrated. In later works, B. Orel et al. reported that mixing barium sulfate (BaSO_4_) with TiO_2_ as pigments in the white paint can improve the cooling performances even further, which is due to the enhanced absorption in the 8–13 μm wavelengths range by the SO_4_ stretching vibrations of the BaSO_4_ content.^32^ Pigmented paints thus may have advantages over polymer films for nocturnal radiative cooling applications due to the flexibility of coating any suitable substrates with easily available regular paints. In further reports, white pigmented paints deposited on different kinds of roof material substrates with 85% solar reflection and 92% broadband IR emission were used for diurnal cooling.^52^ These broadband white paints did not provide cooling below the ambient temperature rather helped to reduce the substrate surface temperature close to the ambient temperature when illuminated under direct sunlight.^52^ In addition to white paints, other type of inorganic materials, such as, SiO films on aluminum film deposited on glass substrates were suggested for efficient radiative cooling.^22^ Utilizing an overlap of intrinsic phonon resonance absorption of SiO and destructive interference for 1 μm thick SiO film, a strong IR emission centered at 10 μm was demonstrated. However, it should be noted that the narrow bandwidth of the SiO emission 20, 21, 22, 24 indicates a small amount of radiated output power within the atmospheric transparency window. Such a narrow bandwidth radiator may not allow substantial cooling as the radiated power can easily be counter balanced by nonradiative heat contributions before the radiator temperature can fall significantly below the ambient temperature. Other types of inorganic materials, such as, silicon nitride (Si_3_N_4_) possessing similar selective emission properties as SiO films were also radiative for potential radiative cooling.^24^ Berdahl et al. reported the potential of magnesium oxide (MgO) and Lithium fluoride (LiF) as selective radiators for radiative cooling and a cooling of 5 °C below the ambient temperature was also demonstrated. The potential of composite materials for high radiance within the 8–13 μm range has also been investigated.^33^, ^42^ Gentle and Smith reported the scope of silicon carbide (SiC) and silica nanoparticles possessing IR emission due to their phonon resonances. The combination of SiC and SiO in multilayers provided even strong, near unity IR emission but spanned beyond the atmospheric window, resulting in a weakly selective IR emitter.^42^ In successive works, it was demonstrated that doping crystalline SiC nanoparticles and SiO_2_ nanoparticles together in polyethylene films deposited on Al can yield high IR emission with better selectivity.^33^ A maximum radiative cooling of 17 °C below the ambient temperature at night was reported in a dry atmospheric condition.^33^

In addition to solid materials, radiative cooling with gases containing selective IR emission was also reported.^48^, ^49^ The first demonstration utilized the highly selective IR radiation of ethylene (C_2_H_2_) gas to demonstrate a maximum cooling of 10 °C below the ambient temperature at daytime but avoiding direct exposure to sunlight. Further reports on radiative cooling by other potential gases such as, ammonia (NH_3_) and ethylene oxide (C_2_H_4_O) were also investigated.^48^ The use of gases for radiative cooling may have potentials; however, the practical application may be challenging as the gases are required to be encapsulated to realize the cooling device. In addition, integration of these encapsulated gases with solar reflectors for radiative cooling under direct sunlight can be more complicated than that with solid radiators.

### 3.2 Solar Reflectors and IR Transparent Convection Cover Materials

To achieve daytime radiative cooling, several kinds of solar reflecting materials were investigated in combination with IR emitters.^25^, ^26^, ^53^, ^54^, ^55^, ^56^, ^57^, ^58^ Among them, Addeo et al. proposed the idea of using IR transparent double layer selective cover for solar reflection where the top layer is highly solar reflective and the bottom layer is poorly solar reflective (highly absorptive).^23^, ^53^ It was estimated that most of the solar radiation can be reflected by the top layer while the small fraction of the solar radiation passing through the top layer can be absorbed by the bottom and an air gap between the cover and the underlying radiator can prevent direct solar heating of the radiator. However, experimental implementation of the selective cover did not provided any practical cooling during the daytime. Andretta et al. employed IR transparent polyethylene film doped with titanium dioxide (TiO_2_) white pigments and carbon black particles as a cover material for the IR radiative emitter.^54^ The pigmented polyethylene films retained high transmittance in the IR wavelengths; however, were not fully reflective within the solar spectra rather contained significant transmission and some intrinsic absorption. Nilsson et al. used zinc sulfide (ZnS) pigmented low‐density polyethylene films for improved solar reflecting foils for daytime radiative cooling.^26^ Although the pigmented films provided high solar reflectance with moderate IR transmittance within the atmospheric window, the foils also contained finite transmission within the solar spectra depending on the volume fraction of the pigments and the thickness of the foils.^26^ In successive works, polyethylene films doped with different pigments, such as titanium dioxide, zinc sulfide, and zinc selenide, possessing improved solar reflectance were demonstrated.^25^ A 400 μm thick polyethylene film including 15% zinc sulfide pigments foil with a high solar reflection of 84.9% and absorption of 13.8% was employed as a cover material for the radiator for daytime cooling. Although significant cooling was demonstrated over the course of the day, no practical cooling was demonstrated at the peak time around noon. In other reports, moderately IR transparent polyethylene films coated with thin semiconductor films (PbS and PbSe) were suggested for use in solar shielding for daytime radiative cooling.^59^ However, the films contained significant transmission in the solar radiation spectral region and reduced the IR transparency to 60%–70%,^59^ leaving the use of these films as potential solar reflectors very challenging. Benlattar et al. proposed the use of cadmium telluride (CdTe) thin film coated silicon substrates as IR transparent solar reflectors; however, the film itself contained 30% solar transmission which can lead to direct solar heating of the IR radiator, making any practical daytime cooling hardly feasible.^45^ Further studies with cadmium sulphide (CdS) films were reported, where, high IR transparency (80%) within the 8–13 μm wavelengths range was demonstrated but no significant solar reflection was achieved.^60^

Apart from solar reflectors, convection covers can also play important role to optimize the performance of the radiative cooler. For daytime applications, if the cooling device's net radiated power (i.e., *P*_r_ − *P*_a_) is higher than the absorbed solar power (i.e., for a cooler with high solar reflectance), the device can yield a temperature well below the ambient temperature provided that convective heat gain is suppressed. On the other hand, if the absorbed solar power is higher than the net radiated power, the device temperature can be higher than the ambient air temperature. In this circumstance, the use of convective covers is undesirable as natural convection can aid to release heat from the cooling device. In the absence of direct solar radiation convection covers can greatly improve the performances for maximum cooling.

In most of the reports, IR transparent polyethylene films were used on top of the radiator with an air gap to provide with a convection shield.^3^, ^10^, ^12^, ^18^, ^50^, ^51^ Polyethylene films are highly transparent in the 8–13 μm wavelengths range with an average transmission over 90% (for an ≈ 10 μm thick film).^10^, ^12^ Alternative cover material with highly IR transparency and mechanical stability, such as zinc sulphide (ZnS), was also suggested.^61^ However, a 4 mm thick ZnS was reported to possess a mean transmittance of only 64% transmission within the 8–14 μm wavelengths range^61^ which can reduce the radiative cooling performance.

## 4 Recent Progress on Highly Efficient and Daytime Radiative Cooling with Photonic Devices

The recent progress on developing radiative coolers based on photonic devices has opened a new window to achieve highly efficient cooling and the ability to operate directly under the sun, reaching temperature below the ambient temperature.^1^, ^3^, ^27^, ^62^ Unlike radiative coolers based on the intrinsic optical properties of bulk materials, these approaches utilize engineered photonic properties combined with the intrinsic properties to realize improved cooling abilities. The unmatched potential of photonic structures is that they can offer strictly selective but strong thermal emission^2^ and very high solar reflection.^3^, ^27^ Different types of photonic devices have been investigated for efficient and daytime radiative cooling. In the following subsections, design principles and photonic characteristics of these devices will be analyzed.

### 4.1 Radiative Cooling with Planar Photonic Devices

**Figure 5:**
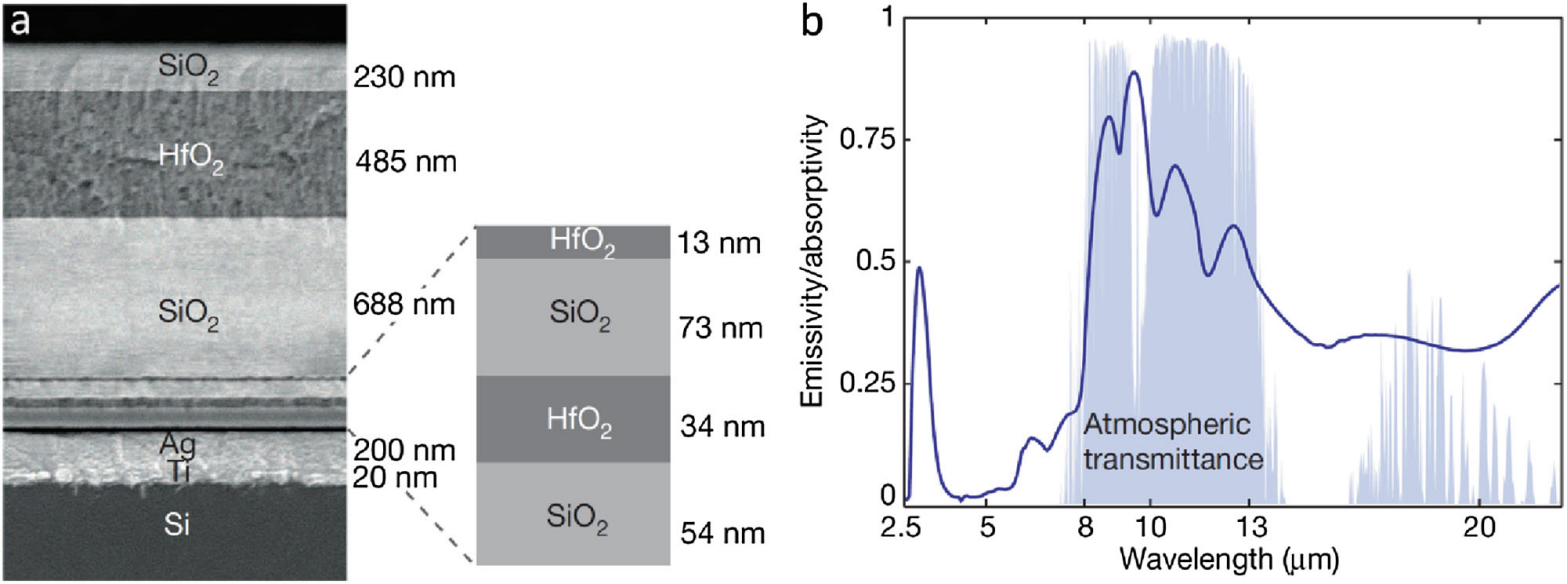
a) Scanning electron microscope image of the radiative cooling device reported in ref. 3. b) The measured emissivity of the radiative cooler at a 5° angle of incidence with the atmospheric window in the background.^3^. Reproduced with permission.^3^ Copyright 2014, Nature Publishing Group.

The first experimental demonstration of radiative cooling with planar photonic devices operating directly under the sun was reported by Raman et al. where, a temperature reduction of 4.9 °C below the ambient temperature was achieved.^3^ The photonic device consisted of seven alternating layers of hafnium oxide (HfO_2_) and SiO_2_ with varying thickness. The top three thick layers mainly contributed to the IR emissivity including the 8–13 μm wavelengths range and the four thinner bottom layers deposited on Al coated Si wafer worked as chirped 1D photonic crystal for a maximum of 97% solar reflection (**Figure** **5**a). With appropriate convective/conductive shielding, the device led to a cooling power of 40.1 W m^−2^ at the ambient temperature even under direct exposure to sunlight, enabling the cooling below ambient temperature during the daytime.^3^ The challenge for this device for achieving further cooling lies between the IR emission properties of HfO_2_ and SiO_2_. First, the IR emission of the device is not strictly selective, which is due to the broadband intrinsic absorption of the materials. Second, the emission within the 8–13 μm wavelengths range is not very strong (Figure 5b). Increasing the thickness of the top three layers may increase the IR emission; however, may also degrade the selectivity of the IR emission. The choice of using the same materials for IR emission and solar restriction has also restricted the flexibility to further optimize the device for improved cooling power.

In another report, Gentle and Smith demonstrated daytime radiative cooling below the ambient temperature by using polymer materials.^27^ Such devices use a stack of alternating layers of birefringent polymers with a refractive index contrast to create a polymer mirror within the solar spectra from 0.4 to 1 μm.^27^, ^63^ To achieve the solar reflection within the IR region, a silver layer was deposited at the bottom of the polymer layers. The polymer films also possessed high but broadband IR emission containing the 8–13 μm wavelengths range. The most appealing result of this device was the demonstrated cooling of 2 °C below the ambient temperature without any convection shield and under a direct 1060 W m^−2^ solar irradiation exposure. In addition, the polymer cooling device provided a cooling of 11 °C below a commercially available white painted roof with a moderate solar reflectivity and IR emissivity.^27^ These results indicate the potential of this polymer device for practical passive cooling application. Further improvement of this cooling device can be made by integrating insulations for nonradiative heat gain. The cooling temperature can also greatly be reduced by replacing the broadband radiator with a strictly selective IR emitter, provided that it is integrated with proper nonradiative insulations. This will be further discussed in Section 5.

### 4.2 Radiative Cooling with Microstructure Photonic Devices

**Figure 6:**
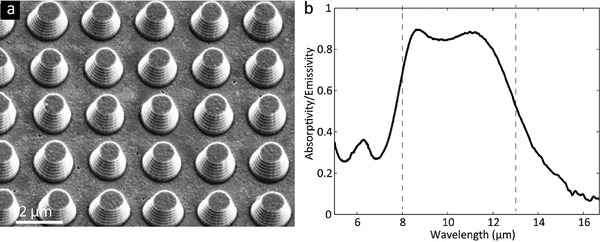
a) The metamaterial microstructure based radiative cooler reported in ref. 2. The measured emissivity of the radiative cooler in ref. 2 for an angle of incidence of 15°–30° with the dashed lines showing the position of the atmospheric window. Reproduced with permission.^2^

The realization of an efficient radiative cooler requires the utilization of strictly selective but strong IR emitter within the 8–13 μm atmospheric window. However, it may be highly challenging to satisfy this condition with the intrinsic IR absorptions in bulk materials and engineered IR absorption within planar photonic devices. Alternatively, highly selective emission can be achieved in artificially designed metallic nano/microstructures, such as, plasmonic structures,^64^, ^65^, ^66^, ^67^, ^68^, ^69^, ^70^, ^71^ metallic photonic crystals,^72^, ^73^, ^74^, ^75^ and metamaterials.^76^, ^77^, ^78^, ^79^, ^80^ However, tuning the emission of these microstructures to achieve selective IR emission covering the entire 8–13 μm wavelengths range can still be a significant challenge. Recent reports demonstrated that some unique metamaterial structures can possess ultrabroadband IR absorptions by utilizing their anisotropy and dispersive properties.^2^, ^78^, ^81^, ^82^ Hossain et al. experimentally demonstrated that these anisotropic metamaterials can be tuned to possess strong and strictly selective IR emission with a near perfect overlap from 8 to 13 μm wavelengths range (**Figure** **6**).^2^ Taking into account the selective IR emission, a projected cooling power of more than 100 W m^−2^ and potential of cooling more than 10 °C below the ambient temperature were estimated.^2^ The application of photonic microstructures toward highly efficient radiative is thus promising; however, can be challenging for practical realization. First, for daytime radiative cooling, the microstructure radiators must be combined with IR transparent solar reflectors to avoid any substantial solar radiation absorption. In practice, this may be difficult, as the currently available planar photonic devices for solar reflection also possess broadband IR emission.^3^, ^27^ Thus, combing these kinds of solar reflectors with microstructure radiators would destroy the strictly selective IR emission. Second, the realization of microstructure radiators often requires lithographic techniques^83^ which can be a challenge for large scale demonstration. However, alternative microfabrication method, such as, UV‐nanoimprint lithography^84^, ^85^ has the potential to provide better feasibility for large scale production.

## 5 Radiative Coolers for Optimized Performance

**Figure 7:**
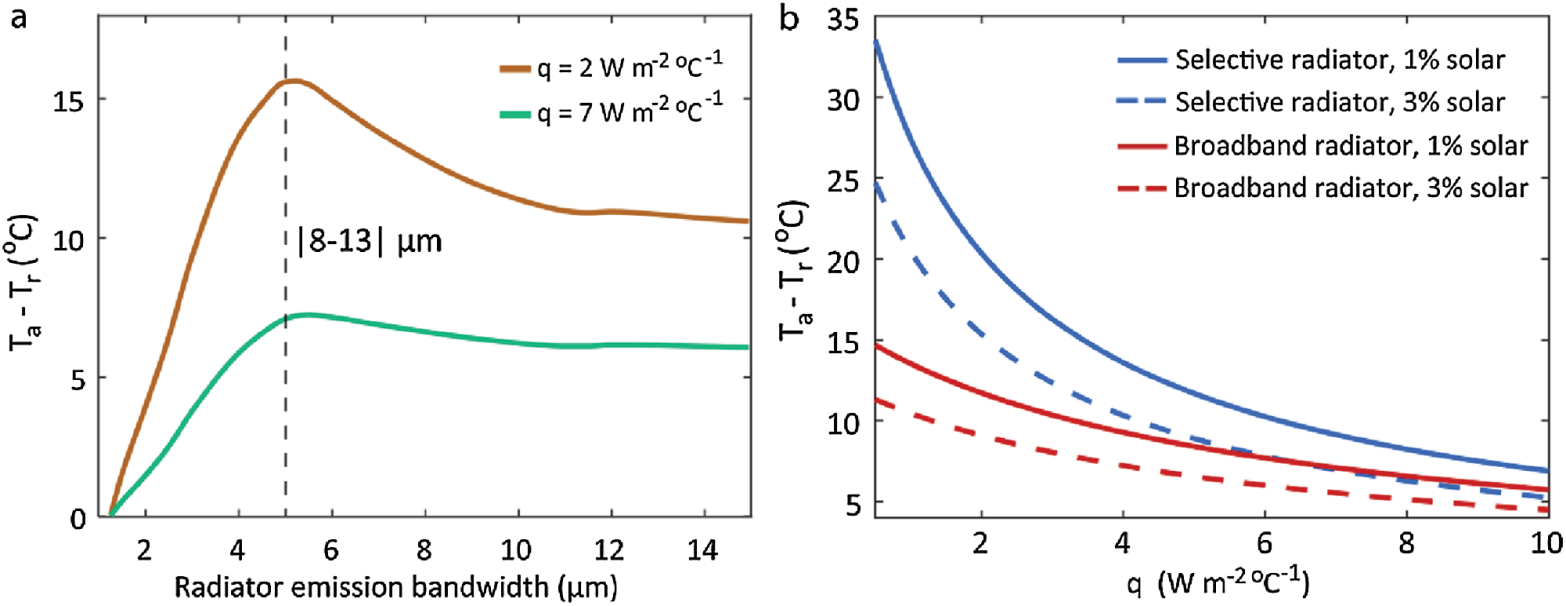
a) Calculated *T*_a_–*T*_r_ for varying the emissivity bandwidth of the radiator with *q* = 2 W m^−2^ °C^−1^ and *q* = 7 W m^−2^ °C^−1^. b) Calculated *T*_a_–*T*_r_ for increasing values of *q* for both selective and broadband radiators. Solid curves represent 1% solar power absorption and dashed curves represent 3% solar absorption.

From the analysis in the previous sections, it is clear that the radiative cooling performance depends on the maximized thermal radiation within the atmospheric window, the radiator emissivity profile, the nonradiative heat gains, solar power absorption, and atmospheric conditions. To further estimate the variation of cooling performance on the emissivity profile of the radiator, we consider a selective radiator with a varying emission bandwidth where the center emission band is at 10.5 μm fitting within the mid of 8–13 μm window range. The atmospheric model for Perth with transmittance within atmospheric window (Figure 4a) is adopted for optimal performance. **Figure** **7**a shows the achievable *T*_a_−*T*_r_ for the variable emission bandwidth including a fixed 3% solar absorption^3^, ^27^ and two high and low values of nonradiative heat gain coefficients. The right side of the vertical dashed line represents the radiators with bandwidth >|8–13| μm of the ideal selective radiator and left side represents the case for bandwidth <|8–13| μm. It is evident that if the cooling device is exposed to a small but practical nonradiative heat gain (2 W m^−2^ °C^−1^),^33^ the ideal selective radiator can provide with a lower cooling temperature (*T*_a_−*T*_r_ > 15 °C) than a broadband radiator. For increasing bandwidth of the radiator, the cooling performance decreases and becomes almost stable for bandwidth >11 μm, where it starts to behave like a blackbody radiator. There is a sudden small increase of the cooling performance for a radiator emission bandwidth >12 μm where the radiator starts to interact with the weak secondary atmospheric window from 16 to 23 μm wavelengths range. For decreasing emission bandwidths, i.e., for bandwidths <|8–13| μm, *T*_a_−*T*_r_ rapidly decreases and does not provide any effective cooling for a bandwidth <1.25 μm. As discussed in Section 2.2, for a radiator with a very narrow emission bandwidth, the small amount of radiated power is unable to offset the solar and nonradiative heat gain.

In the presence of significant nonradiative heat gain (7 W m^−2^ °C^−1^),^3^ the ideal selective radiator does not provide with a notable cooling advantage over the broadband radiator as evident from Figure 7a. We further investigate the achievable *T*_a_−*T*_r_ for both the ideal selective and broadband radiator for varying nonradiative heat gains with a practical 3% solar power absorption^3^, ^27^ and an optimistic value of 1% solar power absorption. It is clear from Figure 7b that for a minimal nonradiative heat gain, the ideal selective emitter can provide with a significantly lower cooling temperature than the broadband radiator. However, the superiority of the selective radiator rapidly diminishes for increasing values of nonradiative heat gain. For *q* = 10 W m^−2^ °C^−1^, the selective radiator does not provide any substantial cooling advantage and the radiator temperature falls only a few degrees below the ambient temperature. Indeed, this points to the recent demonstration of daytime radiative cooling without any nonradiative insulations,^27^ where an ideal selective radiator would not provide with any substantial advantage over the broadband radiator. For atmospheric conditions with higher humidity, the difference in cooling efficiencies for selective and broadband radiators will further be minimized (Figure 4b).

## 6 Conclusion

Radiative cooling promises a vital impact with its highly efficient passive cooling potential. In particular, the recent demonstrations for daytime radiative cooling below the ambient temperature^3^, ^27^ already indicate their ability for practical energy saving applications.^4^, ^5^ A strictly selective but highly emissive radiator with high solar reflectance (≈97%) can deliver a substantial passive cooling during the daytime, leading to temperatures as low as 15 °C below the ambient temperature, provided that insulations for minimized but realistic nonradiative heat gain are employed. Further minimization of nonradiative heat gain and solar power absorption can substantially improve the cooling performance. However, the realization of strictly selective radiators with substantial solar reflection is still a great challenge. Besides, the atmospheric conditions in a particular geographical location will also play a vital role for meaningful radiative cooling efficiency. For applications where integration of nonradiative insulations may not be suitable, broadband radiators can easily replace selective radiators. Furthermore, the cooling efficiency can also be enhanced when radiative cooling can be combined with other passive cooling devices, such as, phase change materials.^86^
